# Polio survivors' perceptions of the meaning of quality of life and strategies used to promote participation in everyday activities

**DOI:** 10.1111/hex.12152

**Published:** 2014-01-20

**Authors:** Anita Atwal, Georgia Spiliotopoulou, Caron Coleman, Kate Harding, Caroline Quirke, Nicole Smith, Zeina Osseiran, Nicola Plastow, Lesley Wilson

**Affiliations:** ^1^ Division of Occupational TherapySchool of Health Sciences and Social CareBrunel UniversityUxbridgeUK

**Keywords:** interventions, participation, polio, quality of life

## Abstract

**Introduction:**

The term ‘post‐polio syndrome’ (PPS) is used to describe new and late manifestations of poliomyelitis that occur later in life. Research in this area has focused upon health status rather than its effect on quality of life.

**Aim:**

To gain an in‐depth understanding of the meaning of quality of life for polio survivors and to determine the type of strategies that are used by people with PPS and the support that they consider as important to facilitate participation in everyday life activities that have an impact on their quality of life.

**Method:**

Six focus groups were conducted with 51 participants from two regions in England. Data were audio‐taped and analysed using thematic analysis.

**Results:**

Our research found that polio survivors used terms used to describe quality of life which could be associated with that of happiness. Our research has identified resolvable factors that influence quality of life namely inaccessible environments, attitudes of health‐care professionals and societal attitudes. Polio survivors have tried alternative therapies, chiefly acupuncture and massage, and found them to be effective in enhancing their quality of life.

**Conclusion:**

It is suggested that health‐care professionals should consider factors which influence happiness and implement a person‐centred approach with the views of the polio survivor being listened to. The three factors that influenced quality of life could be resolved by health‐care professionals and by society. With regard to strategies used, we suggest that polio survivors should have access to the treatments that they perceive as important, although further research is required to design optimal interventions for this client group.

## Introduction and Background

Poliomyelitis (polio) occurs as a result of infection by the polio virus, which usually enters the environment in the faeces of someone who is infected. Persons with non‐paralytic polio usually make a recovery, whilst those with paralytic polio may experience some form of long‐term paralysis. In the 1950s, a vaccine was developed. However, despite its existence and availability, polio outbreaks are still experienced in many developing countries.^1^ Whilst it has been eliminated in most European countries, people with polio are experiencing a new range of symptoms 40–60 years after recovering from paralytic polio. This condition is known as post‐polio syndrome (PPS). It is estimated that 80% of those with polio will experience PPS symptoms. The aetiology for the development of PPS is not clearly known or even fully accepted but the symptoms of PPS include joint and muscle pain, new muscle atrophy, increased muscular fatigability, general fatigue, cold intolerance and bulbar symptoms.^2^, ^3^ Consequently, polio survivors can feel that they have been disabled for the second time.^4^

Quality of life has become a pre‐eminent goal of rehabilitation and a key outcome measure in ascertaining the effectiveness of interventions and rehabilitation programmes. Indeed, maintaining or enhancing quality of life is the ultimate goals of all health‐care professional interventions. Determining the impact of polio on quality of life is central to evaluating treatment efficacy from a service user perspective. A stringent understanding of a patient's quality of life can offer important information regarding the social and personal consequences of disease progression.^5^, ^6^ The physical aspects of PPS have been found to impact upon participation in activities relating to work, leisure social and personal relationships.^7^, ^8^, ^9^, ^10^, ^11^ Conversely, some studies suggest that polio survivors felt that they had lived a satisfactory life despite problems associated with PPS and polio.^12^, ^13^ A contributing factor to this aspect of managing well could be related to the personality of the people who participated in the research since as children polio survivors were encouraged to be active and engage with society.^8^ This has led to the term ‘polio personality’ which is often characterized by over achievement, a strong will to fit in and live a perfectly adequate life.^14^

The differences in these findings could be related to the type of research methods that were used. Within the polio literature, most studies have used surveys and or match controlled trials as opposed to in‐depth qualitative techniques to examine quality of life. This has resulted in fewer avenues for original participant responses, and they were also bound by predetermined definitions for important factors. Each study has used a different set of outcome measures. For instance, Ahlstrӧm and Karlsson^8^ use the quality of life profile which was devised using 120 qualitative interviews. No further information is given about the validity and reliability of the tool. Two other studies used a non‐standardized survey.^15^, ^16^ Atwal *et al*.^16^ analysed two qualitative questions from the survey, whereas Jacob and Shapira's^15^ responses were focused exclusively on activities that had been pre‐selected. Two studies^10^, ^16^ used the Swedish Health‐Related QOL Questionnaire (SWED‐QUAL) which is based on selected measures and consists of 61 items that form 11 multi‐item scales that are aspects of physical, mental, social and general health.

Currently, very little is known about the type of interventions that are used neither by people with poliomyelitis to facilitate their own participation in everyday activities, nor in particular those advocated by core rehabilitation professionals such as occupational therapists and physiotherapists. We conducted a search on MEDLINE (January 1990–April 2012) and found only one paper that evaluated the outcome of physiotherapy with polio survivors.^17^ No papers have evaluated occupational therapy, and no specific pharmacological treatments have been found to be beneficial for the treatment of PPS.^18^ However, despite the lack of evidence, current management is embedded within the management of lifestyle changes, use of proper orthotics and assistive devices, implementing a non‐fatiguing exercise programme, use of physiotherapy and pacing physical activities to prevent muscle and joint overuse.^3^ There is a noticeable lack of research that has explored the effectiveness of interventions from the perspectives of service users. The notable exception is Larsson Lund and Lexell^19^ who aimed to describe and to improve understanding of how people with PPS experienced an interdisciplinary rehabilitation programme. Only 12 people evaluated the programme, but they reported that the rehabilitation programme caused a change in their lives and contributed to the development of new skills. Furthermore, they advocated self‐management as an approach to health care. In addition, Davidson^20^ also evaluated a 9‐day rehabilitation programme but did not collect qualitative data on the users perceptive. They did state that a qualitative study was planned to investigate the patient‐reported benefits of attending the programme.

However, we could not find any evidence that this study had occurred. Moreover, Willén and Grimby^21^ found that from a service user perceptive, a programme of non‐swimming dynamic exercises in heated water has a positive impact on individuals with late effects of polio. However, it was not included in the Cochrane review.^18^

This study aimed to gain an in‐depth understanding of the meaning of quality of life for polio survivors, to determine the type of strategies that are used by people with PPS and the support that they consider as important to facilitate participation in everyday life activities that have an impact on their quality of life.

## Method

To meet the research aims, a qualitative approach was taken and focus groups were chosen as the means of data collection for several reasons. Focus groups enable researchers to understand the perspectives and opinions of a particular group of people by capitalizing on the interaction that occurs in the group setting.^22^, ^23^ For some participants, this may be the first time they had discussed their quality of life and creating a sense of cohesiveness could facilitate people to volunteer information.^24^

Ethical approval was obtained from the University Research Ethics Committee. Six focus groups were conducted with persons with PPS. Four focus groups were held at a university in London. Two groups were held in Leicester at a community centre close to the city centre. Both venues had accessible parking and facilitate to meet the needs of wheelchair users. To ensure that participants who were working could participate, one focus group was run at the weekend. Travel expenses were provided, and all participants received a ten pound gift voucher.

The participants were branch members of the British Polio Fellowship. In total, 300 branch members were approached from the South East and 80 from Leicester. All names were randomly selected from the relevant branch lists. The south‐east of London covers a wide geographical area including London, Surrey and Kent. Leicester is a city in the midlands which is both rural and industrial. A total of 51 people agreed to participate with 35 people attending the four focus groups in the south‐east and 16 attending the two focus groups in Leicester. Each participant attended one focus group only so that the views of all 51 were captured. In our research, the number of participants in each group differed between each group; however, each focus group ranged in size from 7 to 11 participants which is consistent with the focus group literature.^25^, ^26^ Table 1 displays the number of participants along with basic demographics on age and gender in each group from the south‐east and Table 2 from Leicester:

**Table 1:** Demographic characteristics on focus group participants: South‐East

Groups	Number	Gender	Mean age (range) in years
1	8	4 Male/4 Female	65.6 (56–78)
2	11	7 Male/4 Female	66.1 (58–80)
3	9	5 Male/4 Female	65.2 (60–78)
4	7	4 Male/3 Female	63.4 (55–73)

**Table 2:** Demographic characteristics on focus group participants: Leicester

Groups	Number	Gender	Mean age (range) in years
1	7	5 Male/2 Female	67.6 (59–78)
2	9	6 Male/3 Female	73.5 (61–90)

The focus groups were audio‐recorded which not appear to have a restrictive effect on participants in the group. Each group had a moderator, an assistant moderator and an observer, and the members of the research team were given a specific role. In addition, we monitored the group dynamics continuously throughout each focus group session and reflected and discussed them after each focus group. The research team were mindful of the need to facilitate the group but not in a manner which meant that the group was influenced by our own ideas or that we prevented from the group from voicing their own opinion. Hague 27 suggests that in a focus group, the facilitator's voice should constitute between 5 and 10% of the whole transcript. The focus group structure from our research study is outlined in Table 3. The topic guide was used to facilitate discussion, but it was also flexible enough to allow participants to explore issues in depth and allow them to develop themes in their own way.

**Table 3:** Focus group structure

Introduction – explanation of the session and introduction of participants
Discussion of the meaning of quality of life
Activity card sort – Use cards with different definitions of quality of life to prompt discussion about the subjective meaning of the concept for the participants and the impact of polio and post‐polio syndrome on their quality of life
Discussion regarding the types of strategies used to manage pain‐, fatigue‐ and mobility‐related issues. This was written on a whiteboard, and the participants will then discuss the effectiveness of the interventions
Discussion regarding the role of activities within the participants' daily lives and how these impact on QOL, quality‐of‐life priorities and how they do/do not meet these priorities. What support will enable them to meet these priorities
End‐session summary and conclusions

In our research study, we used thematic analysis of the data as suggested by Rabiee,^28^ initially based on group data as the unit of analysis as this is the most common form of analysis.^25^ Before analysing the transcripts, we discussed and reflected upon our own observations from our field notes and our thoughts about how the focus groups had operated. Four researchers worked together to analyse the transcripts and identify major themes and subthemes. We then met to discuss out interpretation. Disagreements were resolved by reaching consensus by jointly rereviewing the transcripts, listening to the audio recordings and discussing the themes at length. The first step in analysis was to become familiar with the data, and this included listening to the focus group recording, reading and rereading of transcripts and field notes, and writing memos of initial ideas about the data.

We carefully ensured that our analysis was sensitive to the various type of interactions that occurred which could be emphatic and challenging, sharing personal experiences and/or talking about others, including humour. We observed that smaller groups ran more smoothly than the larger ones. This could be due to less time constraints which allowed for more extensive individual contributions. The group dynamics seemed to be more challenging in the larger group of 11 participants, where it was harder for more reserved people to participate and therefore some views might have been neglected. A few individuals dominated the conversation, whilst others fairly contributed and very few contributed little or nothing at all despite the facilitator's effort to get views from everyone. Overall, it seems that participants shared a common focus and they were contributing with little encouragement from the facilitator. Personal narratives triggered the discussions and produced empathy and engagement. As the sessions progressed, the participants acted more like members of a group. Sometimes the participants contributed knowledge in a frustrated and irritated way. That was mainly when they were referring to the health‐care system. Participants were often addressing to other fellow participants to seek comfort or compare treatments or symptoms of the disease.

The second stage involved developing themes and sub themes. We systematically worked through the whole of the focus group transcript and labelled chunks of data with a word or phrase that best described the underlying meaning of the text. Data were coded by hand using the ‘long table’. This essentially meant cutting up the transcript and sticking similarly coded extracts together on flip chart paper. We then analysed the data at both a group and an individual level. The aim of this step was to review the initial codes generated with group‐level data, using the individual‐level data.

We first created a separate document for each participant, containing only their individual contribution. Then, we reviewed the group‐level codes in each document to check they were an accurate interpretation for that participant. Finally, we searched across the individual‐level data for new codes that were evident at an individual level, but were not identified in group‐level analysis; we then searched for themes to identify the most prominent themes in the data set. We then reviewed our themes by confirming that the themes were a valid representation of the collective meaning expressed in the group and by ensuring that the themes worked in relation to the whole data set (focus group transcript, audio recording, field notes, memos, codes and their descriptors). In our research, we found that after the 6th focus group, no new issues arose, and thus, we perceived that we had reached data saturation.^24^

## Findings

Four main themes with further subthemes emerged from the focus group analysis. These were meaning of quality of life, important components of quality of life (accessible environments, social belonging and acceptance), knowledge and attitudes of health‐care professionals, coping strategies (living with poliomyelitis, adjusting behaviour, alternative therapies) (see Table 4).

**Table 4:** Themes and subthemes identified through the data analysis

Theme one: Meaning of quality of life
Theme two: Important components of quality of life
Accessible environments
Social belonging and acceptance
Theme three: Knowledge and attitudes of health‐care professionals
Theme four: Coping strategies
Living with poliomyelitis
Adjusting behaviour
Alternative therapies

### Meaning of quality of life

Most of participants in the study perceived that quality of life was impossible to universally define or objectively measure as it was ‘multidimensional’, and contributing factors were ‘different things for each individual.’ Indeed, some participants in focus group 1 concluded that quality of life could only be measured by asking the individual ‘if they had a good quality of life.’ Participants in each focus group disclosed their unique personal concept of the meaning of quality of life which were associated with *‘*being independent,’ ‘feeling good physically and mentally,’ ‘being able to do what you want to do,’ ‘happiness’ and ‘pain free,’ ‘social support from family and friends’ and sufficient ‘heat to keep warm and food.’ Participants in focus group 1 stated that for them it was ‘having choice’, whilst for another participant it was ‘getting up in the morning and I can cycle my bicycle and I'm hoping to God that I can still do that and keep going’.

There were, however, differences between the groups. In focus group 3 and 4, there was the view that your own quality of life may be influenced by how you ‘bench mark your quality of life with people who are disability free.’ Some participants in all of the focus groups emphasized the need of sufficient funds to enjoy a quality of life, whilst ‘Disabled people don't have the income to be able to do exactly what they would like to do.’

## Important components of quality of life

### Accessible environments

Participants in all of the focus group perceived that an accessible built environment and the ability to get around was an essential component of quality of life:

You go into a shop, try and get around the shop and that's another issue, cos they've got so many things in the way, that's a different story, you can't go shopping.

One participant from focus group 5 visited a pub to drink with his friends even though he was not able to access the toilets. In particular, a new mobility aid sometimes meant that access or valued occupations were stopped:

I think I've stopped socialising in the village much. I'm only in a little village and I can't get into my head using the scooter in the village and the little village hall is just too far to walk there and to walk back.

Some of the participants were critical of access to the local swimming pool and or public libraries. One participant in particular reported how her confidence had been decreased gaining access into the swimming pool:

You know these steps down into the swimming pool, they have like pimples on them and stainless steel steps and I slipped through them and it put me right off going again. So I haven't plucked up the courage but I'd like to go swimming.

Participants also expressed concerns about access onto public transport and encountered numerous problems related to getting on/off buses and tubes: ‘public transport to me is a bugbear, a really big bugbear.’ For some participants, their own home environments influenced their quality of life in that they were unable to utilize mobility aids such as electric wheelchairs.

Another participant wanted a bath lift instead of a shower:

I want a bath with a bath lift but they insisted that I have a shower room, so now I have to have someone to help me with it. That type of thing, I'm sure that must affect a lot of people.

Some participants within the focus groups were positive about recent developments in London:

Certainly the built environment does have an influence on ones quality of life, I'm just thinking of some of the new buildings that have been designed.

### Social belonging and acceptance

Participants in focus groups 3 and 4, 5, 6 believed that social belonging and acceptance were important for their quality of life:

Certainly quality of life is social belonging. I think it might be living as closely as we can to what everyone else in society is living. We're not asking for anything over and above, just being able to live as closely as every citizen in the country, they can access everything, enjoy the facilities, engage in work, with people, just doing everything anyone else can do.

Society's attitudes impacted on the lives of persons who attended all of the focus groups, and there was an expressed need to be accepted by members of society:

They fall into two groups. There are those that shove you out of the way, and I have been shoved out of the way sometimes, and there are those that will stand and hold the door for you for half an hour, you know?

Participants in focus group 3 spoke extensively about society's attitude and disregard for people with disability:

Well there still is the element of ‘Does it take sugar?’ syndrome. I've be in a supermarket queues, and sacks of potatoes have been passed over my head, you know things like that, because you know they think you're not in the queue they just seem to think that you are invisible. It does happen a lot when you're in a wheelchair.

Some participants in focus groups 1 and 2 believed that it was ‘about fighting your corner in an able bodied world.’ Many of the participants discussed society's reaction when they were diagnosed as having polio ‘My mum said people wouldn't talk to them in pubs.’ Some participants also recalled challenges that they experienced at school. For some participants in focus groups 1 and 2, there was a sense of relishing their independence and doing the things that mattered to them. Polio survivors perceived that having polio as a child in the 1950s resulted in the participants developing personality traits such as ‘determination’, ‘optimism’, or ‘strong willed’, ‘bolshie’ ‘not asking for any compromises’ that helped them to succeed in life and become ‘survivors’:

I would not be victimised and it made me completely self reliant and not trusting of anybody. I'm still doing it even at 70; I still don't trust other people because all my school life, for 15 years I was brought up not trusting anybody.

## Knowledge and attitudes of health‐care professionals

Health‐care professionals were generally viewed negatively. One participant from focus group 5 stated ‘A physiotherapist was the first person I spoke to in the clinic and he very much dismissed me’, whilst another participant said, ‘Can I say that my doctors are not very accepting?’

Participants in all the focus groups perceived that it was rare for health‐care professionals to research PPS and they were critical about their knowledge of PPS. Furthermore, in focus group 1, some participants avoided seeking medical support as an outcome of their negative experiences with professionals during the acute episode of polio and recent late onset of PPS. Participants in focus groups 3 and 4 expressed their frustration about time spent educating health‐care providers about polio: ‘Most of the anaesthetists wouldn't know what to do if you just turned up in hospital.’ Many participants in these groups generally agree that polio is considered a ‘dead disease’ a thing of the past by health‐care professionals because it has been eradicated and that this influences health‐care professional's attitude to understanding and treating people with polio.

The message from the participants in all 6 focus groups was similar: ‘they ought to listen to us rather than we listening to them.’ Another participant from focus group 5 stated that ‘professionals don't take notice if you are disabled, they think you are dumb.’ One participant in focus group 6 was aware of what he needed, but this was not listened to as *‘*professional people don't like unqualified people telling them their own work.’

Thus, some of the participants in focus group 2 and 6 were critical of professionals for delivering treatments to the PPS population without knowing their effectiveness. There was also a sense that GPs did not know what to do and participants frequently experienced the response: ‘what do you expect me to do about it?’ Indeed, participants in all focus groups explained that they had received various treatments which had been ineffective and inappropriate:

And it is about trial and error with them because go away and take this and then a couple of years later you've got something else wrong with you because of the side effects.

Participants in focus group 3 perceived that standard tests are not capturing the necessary information to make an appropriate judgement about their symptoms. Participants in focus groups 5 and 6 perceived that their GPs were reluctant to refer them for scans or tests or physiotherapy. Although pain ‘all the time’ was reported by many of the participants in all of the focus groups, they perceived that the options to managing this pain were not ‘acceptable’ particularly in relation to medication. In some instances, participants reported that the GPs poor prescribing had severely impacted on their health and wellbeing:

I was prescribed the wrong medication and one of the side effects was to get swelling, unexplained swelling. I recently went to the Doctor and I asked him what is the swelling for and he didn't tell me and then I said look it's because I had done some research and I found out. He couldn't look me in my eyes at the time because he knew that he had given me wrong medication.

For some participants, there seems to be a desire to prove health‐care professionals wrong:

I was told by the physiotherapist in the hospital that I would never, ever be able to use my arm again, and that made me so angry. At thirteen and a half I was absolutely furious, I wanted to punch her, and I have used my arm and I've had three children, three sons. I have not been able to carry them in my arm but I've done everything else for them. I've carried them in my left arm, and I have nothing to complain about.

## Coping strategies

### Living with poliomyelitis

Maintaining a positive mind by being positive was an essential strategy for participants in focus groups 1 and 2 to avoid low mood and or depression:

It's also about you've got a choice, you've got choice in life. Every day, when you wake up, you can get up and say, ‘I'm depressed, I hate the world, I hate this….’ or you can just simply get up and take the more positive route. Which I think the majority of the people around this table do.

Participants in these groups reported that they sustained a positive outlook by not worrying about things they could not change or as one person said ‘the meaning of life is that it has to be lived.’ Humour was also used by some of the participants in focus groups 2, 5 and 6. Several focus group 1 participants agreed that they strived to achieve daily goals, in spite of low motivation. Some participants in focus group 3 were in agreement that it is difficult to stay positive after having PPS describing it as ‘unfair’ and ‘worse than the actual polio.’

For some participants in focus groups 3 and 4, pain was managed by ‘living with it’ or ‘simply getting on with it’ or as ‘mind over matter’ as ‘this pain isn't going to kill us and it becomes just a nuisance.’ For other participants, pain is a ‘fact of life.’ However, for some participants, they appeared to regard as not managing the pain as failing as individuals ‘You blame yourself, you feel like, you should be stronger to face the pain in order to be able to do what you had in our diary. You really start picking holes in yourself.’ Some participants in focus group 2 reported that doing ‘the classic polio thing’, trying to ‘over achieve’ and ‘do too many things’ exacerbated PPS symptoms.

### Adjusting behaviour

Many of the participants in all six focus group reported feeling fatigued and tired which impacted upon their participation in chosen occupations such as walking and shopping. Participants in all focus groups reported that increased fatigue often disrupted their plans which restricted their ability to engage in different social activities such as snooker or bridge.

However, some participants had now chosen to participate in less physical demanding activities like activities like egg decorating, dominoes league, quizzes, ceramics courses and TV, as these activities still offer a mental challenge but did not require much physical exertion.

Numerous individual strategies were used by the participants who often focused around having a routine or allowing extra time:

Well, I do find it difficult to do things more and more, so I have to compromise in a different sort of way. Even when I'm cooking, my oven is low and I'm in a small electric wheelchair so what I have to do is take the stuff out, use the elbow I know that sounds weird but I push the thing with my elbow to turn it and put it onto something and then lift it up, so I have to compromise with everything I do. I still do it but it takes me a long time and I have to be very careful.

Pacing was viewed as an important strategy in all six focus groups:

When there are things to be done in the house be it of a practical nature or a paper nature, I use a method called “just a bite at a time.” The trouble is the “just a bites” never finish.

### Alternative therapies

Participants in most focus groups had tried or still used different types of alternative therapies including acupuncture, reflexology, osteopathy and massage. In focus groups 3 and 4, there was disagreement about the effectiveness of acupuncture in managing fatigue. Participants in focus groups 5 and 6 had also tried transcutaneous electrical nerve stimulation (TENS) with differing degrees of success to manage pain and also hot baths and heat. Participants in focus group 2 felt that a limiting factor was that the health service in England does not pay for treatment which would benefit them. Most participants in focus groups 2, 3, 4, 5 and 6 perceived massage, hydrotherapy and acupuncture to be expensive, although highly effective. There was also the view from participants in focus groups 3 and 4 that money could buy you the best intervention:

when you have pain, like we all have and you want to feel better, if you haven't got the money you put up with it.

## Discussion

Our research is the first qualitative study to explore polio survivors perceptions of the meaning of quality of life and strategies used to promote participation in everyday activities. Our research found that persons with PPS perceived that it is difficult to define quality of life as it was perceived as being subjective and multidimensional. This is acknowledged within the World Health Organization 29 definition of quality of life which is generally adopted by health‐care providers and professionals. In addition, our research found that the terms used to describe quality of life are associated with that of happiness. This is a new finding and has not been reported elsewhere in the polio literature. Happiness can be viewed as three components (enjoyment, satisfaction and excellence) which are all separate variable of happiness.^30^ Polio survivors described quality of life in relation to these components, for example. enjoyment, for example ‘feeling good physically and mentally’, ‘satisfaction’ being ‘able to do what you want to do’ excellence in relation to good quality activities ‘getting up in the morning and I can cycle my bicycle.’ Thus, for polio survivors, an open‐ended question that incorporates the components of happiness may be more meaningful than an actual quality‐of‐life measure. Indeed, there has been much debate as to how happiness can be measured.^31^ Moreover, there was view by some that polio survivors should not be bench marking their quality of life with people who were ‘disability free.’ Therefore, studies that have used match controls to explore life satisfaction or fatigue with polio survivors (see^7^, ^32^, ^33^) may be perceived as having little value by polio survivors.

Our research has identified various barriers that impact upon a person's quality of life and participation in everyday activities. We suggest that many of these factors are avoidable and could be resolved by health‐care professionals and society. The first barrier is in relation to participation in chosen activities was in relation to environmental issues, and our research highlights their daily struggles with the physical environment. This in turn could result in polio survivors not having ‘equal access’. Inaccessible built environments impacted on polio survivors’ participation in participation in activities such as work^13^, ^34^ and autonomy outdoors^9^, ^13^ and participation in chosen social activities.^16^ Issues with transport have also been previously noted by Burger and Marinček^9^ and Atwal *et al*.^16^

Attitudes were another barrier which impacted on quality of life from both health care, societal, family and friends and professionals. It is surprising that there no explicit reference to carers even though some of the participants made some reference to the importance of friends.

Societal attitude was another barrier to participation in activities. This is a new finding and has not been reported within the polio literature, although in disability research societal attitudes towards persons using wheelchairs are well documented.^35^, ^36^ In addition child hood experiences of living with polio may account as to why polio survivors continued battle to be accepted within an able bodied world. This finding is supported by the work of Wenneberg and Ahlstrom^12^ and Hollingsworth *et al*.^37^ who report that as children polio survivors’ self‐image was damaged by being brutally exposed to other people's prejudices about polio and disability. Indeed Yelnik and Laffont^38^ make similar observations after reflecting upon their own clinical experiences of managing patients with PPS. Thus, this may account as to why some polio survivor's strategy to enable participation in chosen activities was simply to ‘get on with it’.

Our research supports previous research^2^, ^14^, ^16^ that health‐care professionals did not understand and or have the knowledge to manage PPS and polio, although our research also suggests that mistrust between health‐care professionals and patients appears to have developed over time which may have impacted on polio survivors seeking help. Similar findings have also been reported in a survey conducted by Calvert *et al*. 39 with people with rare long‐term neurological conditions where a subsample of 40 patients with PPS was used. This study demonstrated that although some patients have used rehabilitative services, the care co‐ordination needs further improvement to improve health‐related quality of life. Polio survivors described instances where their symptoms had been ‘miss managed’; indeed, being ‘pain free’ was described within the definition of quality of life. Previous research has described pain as a factor impacting upon participation in activities.^40^, ^41^ In other instances, polio survivors described instances when professionals did not ‘listen’. Further research is needed to explore how health‐care professionals implement the concepts of patient‐centred care and more importantly the need to explore further the concept of patients being experts within their own fields.

Polio survivors in our study were able to clearly articulate their views about what would enhance their quality of life, particularly in relation to the treatments that are available. Our study found that many people with PPS have tried alternative therapies, chiefly acupuncture and massage, and found them to be effective in enhancing their quality of life. This has not been reported in the previous literature; however, it must be noted that a Cochrane systematic review^42^ concluded that massage therapy may effectively manage non‐specific lower back pain. Numerous strategies were used by polio survivors. In our research, some polio survivors had changed their lifestyle using diet, exercise, hydrotherapy and pacing (energy conservation). Whilst a Cochrane review found little evidence to support their use,^18^ a qualitative study by Larsson Lund and Lexell^19^ found that PPS participants found that energy conservation managed fatigue and reduced pain. In addition, some studies have reported the benefits of community‐based aquatic exercise programmes for increasing quality of life.^43^

### Limitations of the study

In our focus groups, we needed to ensure that carers did not contribute to the group and/or speak on behalf of group members. The composition of the group can impact upon the type and amount of interaction that occurs in the group and in some of the focus groups contained more men than women. It would have also been useful to have gathered information on the occupation and marital status of the participants, as this could have informed further the interpretation of the findings. It is suggested that researchers should ensure ‘commonality’ not diversity within focus groups.^44^ Our focus groups consisted of people with PPS; however, the way the symptoms have impacted upon an individual's health and well‐being varies from each individual. Moreover, as we wanted to be inclusive, we did not want to exclude anyone because of their gender, social or educational characteristics. We did, however, take some of these factors into account when we analysed the data.

## Conclusion

The findings from our research suggest that it would be beneficial to allow individuals to select the dimensions that are of most concern which relate to their quality of life. Our research found that polio survivors used terms used to describe quality of life which could be associated with that of happiness. It is suggested that health‐care professionals should consider factors which influence happiness. Health‐care providers and professionals need to ensure that they take a person centred approach with the views of the expert (polio survivor) being listened to. Our research has identified factors that influence quality of life, namely inaccessible environments, attitudes of health‐care professionals and societal attitudes. These are resolvable, and any rehabilitation programme need to ensure that these issues are discussed and strategies out in place to enable participation in chosen activities. With regard to strategies used, we suggest that people with PPS should have access to the treatments that they perceive as important, although further research is required to design optimal interventions for this client group.

## Sources of funding

None.
